# Clinical Decision-Making in the Treatment of Schizophrenia: Focus on Long-Acting Injectable Antipsychotics

**DOI:** 10.3390/ijms17111935

**Published:** 2016-11-19

**Authors:** Ludovic Samalin, Marion Garnier, Candy Auclair, Pierre-Michel Llorca

**Affiliations:** 1 Centre Hospitalier et Universitaire, Department of Psychiatry, University of Auvergne, EA 7280, 63000 Clermont-Ferrand, France; lsamalin@chu-clermontferrand.fr (L.S.); m_garnier@chu-clermontferrand.fr (M.G.); 2 French Society for Biological Psychiatry and Neuropsychopharmacology, CH Sainte Anne, 75674 Paris, France; 3 Fondation FondaMental, Hôpital Albert Chenevier, Depatment of Psychiatry, 94000 Créteil, France; 4 Centre Hospitalier et Universitaire, Department of Public Health, University of Auvergne, EA 4681 PEPRADE, 63000 Clermont-Ferrand, France; cauclair@chu-clermontferrand.fr

**Keywords:** long-acting injectable, antipsychotic, decision-making, guidelines, schizophrenia

## Abstract

The purpose of this study was to identify clinician characteristics associated with higher prescription rates of long-acting injectable (LAI) antipsychotics, as well as the sources that influence medical decision-making regarding the treatment of schizophrenia. We surveyed 202 psychiatrists during six regional French conferences (Bordeaux, Lyon, Marseille, Nice, Paris, and Strasbourg). Data on the characteristics of practice, prescription rates of antipsychotic, and information sources about their clinical decisions were collected. Most psychiatrists used second-generation antipsychotics (SGAs), and preferentially an oral formulation, in the treatment of schizophrenia. LAI SGAs were prescribed to 30.4% of schizophrenic patients. The duration and type of practice did not influence the class or formulation of antipsychotics used. The clinicians following the higher percentage of schizophrenic patients were associated with a higher use of LAI antipsychotics and a lower use of oral SGAs. Personal experience, government regulatory approval, and guidelines for the treatment of schizophrenia were the three main contributing factors guiding clinicians’ decision-making regarding the treatment of schizophrenia. The more clinicians follow schizophrenic patients, the more they use LAI antipsychotics. The development of specialized programs with top specialists should lead to better use of LAI antipsychotics in the treatment of schizophrenia.

## 1. Introduction

Schizophrenia is a chronic illness with a high risk of relapse that is frequently associated with treatment discontinuation. Ensuring treatment adherence is one of the principal challenges in schizophrenia management. This requires awareness of several risk factors (e.g., symptoms of the illness, stigmatization, poor therapeutic alliance, and a complex medication schedule) [1].

The development of long-acting injectable first-generation antipsychotics (LAI FGAs) in the 1960s and, more recently, of long-acting injectable second-generation antipsychotics (LAI SGAs) has been an important step in the management of schizophrenia. These formulations are considered as one of the most efficacious pharmacological interventions available to address adherence problems in patients suffering from schizophrenia [2,3]. They offer several advantages, including transparency of adherence and the possibility of early intervention of healthcare professionals if patients fail to take their medication, as well as, from a pharmacokinetic perspective, more consistent bioavailability and reduced peak-trough plasma levels [2,4].

LAI FGAs and LAI SGAs have been shown to be effective in the long-term treatment of schizophrenia, with the specific impact of reducing the risk of relapse [4]. While the efficacy of LAI antipsychotics compared with a placebo in randomized clinical trials (RCTs) is well established, the evidence for the specific advantages for LAI SGAs over oral medication remains unclear [5,6]. The effectiveness of LAI antipsychotics has been debated due to some inconsistent findings in the literature in comparison to results of oral formulation treatments. The benefits of LAI antipsychotics are significant in observational or mirror-image studies; however, they failed to reach statistical superiority in RCTs [3,7]. Study design seems to be an important factor. One possible explanation is that RCTs do not reflect the reality of clinical practice (patients who consent to participate are willing to be more involved in the care process and have better adherence) [7].

For a long time, the use of LAI antipsychotics has been reserved for patients with chronic illness and poor adherence. However, recent guidelines for the use and management of LAI antipsychotics in clinical practice evoke the possibility of using this formulation for patients with first-episode or recent-onset schizophrenia [8,9,10,11,12]. Although most clinicians regard them as effective, reported LAI antipsychotic prescribing rates are low [13] and vary widely between countries (6%–30%) [14]. This highlights the barriers that exist to the prescription of these formulations.

We hypothesize that there are some characteristics in clinicians’ practice that might be associated with different modalities of prescription of LAI antipsychotics. The aim of our study was to identify (i) the characteristics of practice associated with higher LAI antipsychotic prescription rates; and (ii) the sources of information that influence medical decision-making regarding the treatment of schizophrenia.

## 2. Results

### 2.1. Participants

A total of 202 psychiatrists participated in the survey (Table 1). The mean age and career duration were 43 ± 11 years and 13 ± 11 years, respectively. The majority of clinicians treated outpatients and inpatients. Almost half of the patients followed by clinicians (46%) were treated for schizophrenia. Clinicians declared that they prescribed oral SGAs to more than half of their patients and LAI SGAs to 30% of their patients.

### 2.2. Modalities of Prescription according to the Practice Characteristics of Clinicians

Table 2 shows the correlations between the type of antipsychotic used and the practice characteristics of clinicians. The class and the formulation of the antipsychotics were not influenced by the duration of the clinicians’ career or their type of practice. The higher the percentage of schizophrenic patients followed by clinicians was, the more LAI antipsychotics (FGAs and SGAs) and fewer oral SGAs were used.

### 2.3. Modalities of Decision-Making about Treatment of Schizophrenia

**Figure 1:**
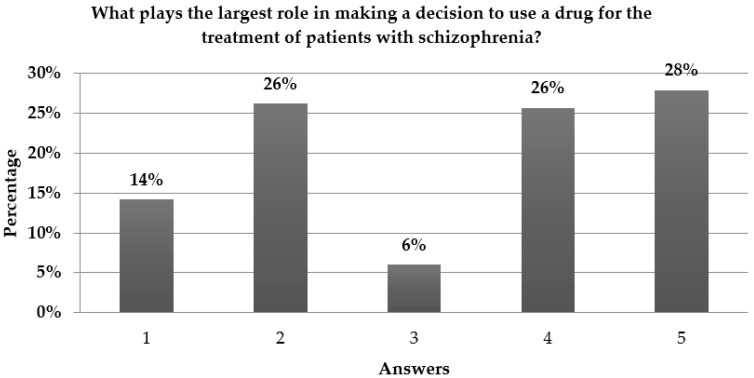
Factors influencing the medical decision-making regarding treatment of schizophrenia. Answers: 1. Published clinical study literature; 2. Guidelines for the treatment of schizophrenia; 3. Information from congresses or conferences; 4. Government regulatory approval; 5. Personal experience.

Personal experience, government regulatory approval, and guidelines for the treatment of schizophrenia were the three main contributing factors guiding clinicians’ decision-making regarding treatment of schizophrenia (Figure 1). Published clinical studies only played the largest role as the primary source in the selection of appropriate therapy for 14% of clinicians, while information from congresses or conferences played the largest role for 6% of clinicians.

There were some differences in the factors influencing the clinical decision-making of psychiatrists about treatment according to their practice characteristics (Table 3). Personal experience, as the main factor guiding clinical decision-making, was significantly associated with a higher career duration of clinicians (*p* = 0.029). The use of guidelines for the treatment of schizophrenia was significantly associated with a higher proportion of schizophrenic patients in the follow-up of clinicians (*p* = 0.049).

## 3. Discussion

In this naturalistic sample of French psychiatrists, practitioners declared that more than 30% of their treated schizophrenic patients received LAI SGAs.

Specifically, our findings showed the following:

- Clinicians treating the highest proportion of schizophrenic patients prescribed significantly more LAI antipsychotics and fewer oral SGAs.
- Other clinicians’ practice characteristics were not significantly associated with a differential use of LAI antipsychotics.
- Personal experience, government regulatory approval, and guidelines for the treatment of schizophrenia were the main factors guiding clinicians’ decision-making regarding the type and formulation of antipsychotic prescribed.

Career duration appeared to be associated with decision-making regarding treatment based on the personal experience of psychiatrists. Conversely, the proportion of schizophrenic patients seemed to be associated with evidence-based decision-making on the part of clinicians (using guidelines for the treatment of schizophrenia).

Psychiatrists reported prescribing SGAs (oral and LAI) for most of their schizophrenic patients, as recommended as first-line treatment by international guidelines for the management of schizophrenia [15]. The prescription rate of LAI formulation appeared higher in comparison with other recent French studies (10%–15%) [16,17]. There is a possible lack of correspondence between the prescription rates reported and every day practice (as described by a study on French prescription [17]). Psychiatrists probably overestimated their real use of LAI antipsychotics in clinical practice.

The LAI antipsychotic prescription rate of varies widely in other countries around the world (e.g., United Kingdom 28%–36%, Belgium 21.5%, Australia 25%, New Zealand 15%, United States 29%, and China 23%) [18,19]. The health care system (e.g., the cost of drugs, accessibility, and specialized psychiatric hospitals) and the training of psychiatrists about the use of antipsychotics differ between countries and could be a factor explaining these differences. However, these cultural aspects are not sufficient to explain, whatever the countries, why the LAI antipsychotic prescription rate is generally lower than 30% in patients with schizophrenia.

Among the practice characteristics of psychiatrists involved in this study, only the proportion of schizophrenic patients was significantly associated with the prescription of LAI antipsychotics. Clearly, the higher the percentage of schizophrenic patients followed by clinicians, the more LAI antipsychotic formulations were used. Previous studies have shown that clinicians with more experience of LAI antipsychotics provided more information about LAI antipsychotics to their patients and had more favorable attitudes toward LAI antipsychotics than psychiatrists with less experience [20,21]. They also obtain significantly higher LAI antipsychotic acceptance rates in patients [21]. On the other hand, factors such as limited knowledge and experience with LAI antipsychotics, negative attitudes toward them, and prescribing practices reducing the use of them to a “last-resort” for patients with a past history of non-adherence appear to limit the use of LAI antipsychotics by psychiatrists [22]. This suggests that there is probably a virtuous circle between the level of experience in treating schizophrenic patients, the level of experience in LAI antipsychotic use, and attitudes toward LAI antipsychotics regarding the prescription rate of these formulations.

There was no significant correlation between the type of antipsychotic used (class or formulation) and the other characteristics of clinicians (age, duration of career, and type of practice). In contrast, a previous German study showed that “older” psychiatrists offered and prescribed significantly more LAI FGAs and fewer LAI SGAs than did their “younger” colleagues [23]. A possible explanation for this is that the recent development of LAI SGA formulations, offering numerous equivalences with oral SGAs (e.g., olanzapine, risperidone, paliperidone, and aripiprazole), has facilitated their use independently of the age of clinicians or their career duration, due to an improved benefit–risk ratio compared with LAI FGAs.

It is worth mentioning that factors influencing the decision-making about the treatment of schizophrenia were mainly a combination of experience and evidence-based medicine. In comparison with a previous French study, we noted a steep increase in the use of guidelines in clinical practice from 12.5%, in 2007–2008, to 26%, in the present study [24]. One hypothesis could be that the recent development of specific French guidelines for the use and management of LAI antipsychotics in serious mental disorders [11,25], using a consensus-based methodology involving practitioners, facilitated their adherence and their use in clinical practice. The primary reasons given for the non-use of guidelines by French psychiatrists were related to the cultural differences between Anglo-Saxon practice and French practice, and were also related to the distinction between expert and practitioner psychiatrists [24]. Moreover, the implementation of educational regional workshops in France following their publication probably also helped to promote their use, as highlighted by a recent Cochrane systematic review [26].

The level of experience in treating schizophrenic patients also appeared to be significantly more correlated with the use of treatment guidelines. Though guidelines have been established to help clinicians choose appropriate care in specific clinical circumstances, most of them have been developed for specific psychiatric disorders (e.g., schizophrenia, bipolar disorder, and anxiety disorders). Clearly, general psychiatrists cannot do not have the time necessary to read all of the different guidelines (and their updates) from the different agencies or societies for all the serious mental disorders. Consequently, the fact that more specialized psychiatrists in schizophrenia used guidelines for the treatment of schizophrenia in clinical practice more frequently makes sense.

These points highlight the need to develop specific strategies to provide optimal care for patients with schizophrenia. In the United States (Texas), the potential solution was the creation of the A ShoT At Recovery (A-STAR) program. In this organization, the aim of the integrated team is to enhance the use of LAI medication by providing data and education, both to prescribers and to patients [27]. In France, the development and implementation of the Schizophrenia Expert Centers (Fondation FondaMental) is meant to have the same impact. Clinicians working in this organization develop a hyper-specialization in treating schizophrenia. The goal of this network is to promote an evidence-based but personalized medicinal approach, and to provide the best therapeutic strategies for each patient to obtain remission and reduce the risk of relapse [28].

The main limitation of this study is the cross-sectional design that did not allow analysis of the causal relationships between the type of antipsychotic used and the practice characteristics of clinicians. Another relevant issue is that the participants may not necessarily be representative of all French psychiatrists due to a possible selection bias. Gender distribution of the participating psychiatrists was close to the national values published by the French National Medical Council (50% males, 50% females); however, our survey involved psychiatrists younger than the national mean age (43 vs. 51 years old) [29]. Finally, despite recent published data about the LAI antipsychotic prescription rate in France [17] based on global data from the national insurance system, the design of our study does not make it possible to match the participants’ prescription rate in their everyday practice and what they report.

## 4. Materials and Methods

### 4.1. Procedure

We conducted a cross-sectional survey among psychiatrists during six regional conferences (Bordeaux, Lyon, Marseille, Nice, Paris, and Strasbourg) of the French Society for Biological Psychiatry and Neuropsychopharmacology between December 2014 and April 2015.

We identified a randomized sample of 250 participants from the global list of participants (approximately 500). Recruitment of clinicians took place during each conference. All subjects participated with informed, voluntary, and written consent.

The procedures followed in the study were approved by an independent national ethics committee (CPP Sud-Est 6) and were conducted in accordance with the revised version of the Helsinki Declaration (1989).

### 4.2. Assessments

A self-administered questionnaire was completed anonymously and returned at the end of conference by each participating psychiatrist.

The questionnaire comprised three sections:

- demographic characteristics and characterization of practice (i.e., gender, age, duration of career, type of practice, and proportion of schizophrenic patients follow-up);
- estimation of antipsychotic prescription rates by the psychiatrists (oral FGAs, oral SGAs, LAI FGAs, and LAI SGAs);
- sources of information influencing the medical decision-making regarding the treatment of schizophrenia—clinicians had to grade the sources of information proposed (i.e., published clinical studies, guidelines for the treatment of schizophrenia, information from congresses or conferences, government regulatory approval, and personal experience) from 1 (first-line source) to 5 (last-line source).

### 4.3. Statistical Analysis

Means and standard deviation were calculated for continuous measures, and categorical data were presented as frequencies. Correlation analyses of the type of antipsychotic used in schizophrenic patients and practice characteristics of clinicians were performed using Spearman correlations. Inter-group comparisons, according to the sources of information influencing decision-making about treatment, were performed using the Wilcoxon–Mann–Whitney test.

Statistical analyses were performed using SAS 9.3 software (SAS Institute Inc., Cary, NC, USA). All statistical tests were two-tailed, and the significance level was set at 5%.

## 5. Conclusions

Despite recent evidence showing the benefits of the use of LAI antipsychotics early in the course of the management of patients with schizophrenia, the gap in their use in clinical practice has remained wide. Numerous barriers, including the overestimation of patients’ adherence, patient refusal, or perceived coercion, have been identified. However, no effective strategies have reduced these barriers or changed the prescribing behavior of psychiatrists. Our findings highlight the significance of the current development of Expert Centers and other programs specialized in the management of schizophrenic patients and of patients with other serious mental disorders across the globe.

## Figures and Tables

**Table 1:** Sample of participating psychiatrist (*N* = 202).

Characteristics of Psychiatrists	*N* (%)
Gender (males)	97 (48.0)
	**Mean ± SD**
Age (years)	42.8 ± 11.4
Duration of career (years)	13.0 ± 10.7
Type of practice (%)	
Outpatient	55.4 ± 29.1
Inpatient	60.2 ± 27.2
Proportion of schizophrenic patients followed (%)	46.3 ± 24.3
Prescription of antipsychotics (% of schizophrenic patients)	
Oral FGAs	16.2 ± 13.2
Oral SGAs	54.9 ± 21.7
LAI FGAs	13.6 ± 12.2
LAI SGAs	30.4 ± 19.3

FGA: first-generation antipsychotic; LAI: long-acting injectable; SGA: second-generation antipsychotic.

**Table 2:** Correlations between type of antipsychotic used in schizophrenic patients and practice characteristics of clinicians.

Antipsychotics	Career Duration (Years)		% of Inpatients		% of Schizophrenic Patients	
	r	*p*-Value	r	*p*-Value	r	*p*-Value
Oral FGAs	0.10	0.23	0.04	0.60	0.06	0.41
Oral SGAs	0.04	0.65	−0.07	0.33	−0.25	**<0.01**
LAI FGAs	0.11	0.16	0.01	0.94	0.35	**<0.01**
LAI SGAs	0.04	0.62	0.05	0.51	0.27	**<0.01**

FGA: first-generation antipsychotic; LAI: long-acting injectable; SGA: second-generation antipsychotic; r: Pearson correlation coefficient. Bold values are *p* < 0.05.

**Table 3:** Decision-making regarding treatment according to the practice characteristics of clinicians.

Factors Affecting Decision-Making		Career Duration, Mean (Year) (SD)		% of Inpatients, % (SD)		% of Schizophrenic Patients, % (SD)	
			*p*-Value		*p*-Value		*p*-Value
Published clinical study literature	Yes	11.1 (10.2)	0.47	41.2 (35.6)	0.64	38.0 (25.7)	0.09
	No	12.7 (10.6)		50.3 (32.6)		47.2 (23.9)	
Guidelines for the treatment of schizophrenia	Yes	10.2 (9.6)	0.11	46.7 (30.4)	0.64	52.0 (23.0)	**0.05**
	No	13 (10.8)		49.8 (33.9)		43.5 (23.5)	
Information from congresses or conferences	Yes	16.1 (13.0)	NA *	51.0 (38.7)	0.86	28.9 (24.2)	NA *
	No	12.3 (10.4)		49.1 (32.7)		47.1 (24.0)	
Government regular approval	Yes	11.4 (9.0)	0.68	48.3 (35.7)	0.89	49.4 (25.4)	0.36
	No	12.9 (11.1)		49.4 (31.9)		45.0 (23.9)	
Personal experience	Yes	15.4 (11.8)	**0.03**	55.1 (30.4)	0.13	44.1 (22.0)	0.61
	No	11.3 (9.8)		46.5 (33.7)		46.5 (25.3)	

* NA: statistical test not applicable. Bold values are *p* < 0.05.
